# Analysis of Consistency in Emergency Department Physician Variation in Propensity for Admission Across Patient Sociodemographic Groups

**DOI:** 10.1001/jamanetworkopen.2021.25193

**Published:** 2021-09-21

**Authors:** Hazar Khidir, J. Michael McWilliams, A. James O’Malley, Lawrence Zaborski, Bruce E. Landon, Peter B. Smulowitz

**Affiliations:** 1 Harvard Affiliated Emergency Medicine Residency, Boston, Massachusetts; 2 Now with Yale School of Medicine, New Haven, Connecticut; 3 Department of Health Care Policy, Harvard Medical School, Boston, Massachusetts; 4 Department of Biomedical Data Science, The Dartmouth Institute for Health Policy and Clinical Practice, Geisel School of Medicine at Dartmouth, Hanover, New Hampshire; 5 Division of General Internal Medicine, Beth Israel Deaconess Medical Center, Boston, Massachusetts; 6 Department of Emergency Medicine, University of Massachusetts Medical School, Worcester; 7 Milford Regional Medical Center, Milford, Massachusetts

## Abstract

**Importance:**

Sociodemographic disparities in health care and variation in physician practice patterns have been well documented; however, the contribution of variation in individual physician care practices to health disparities is challenging to quantify. Emergency department (ED) physicians vary in their propensity to admit patients. The consistency of this variation across sociodemographic groups may help determine whether physician-specific factors are associated with care differences between patient groups.

**Objective:**

To estimate the consistency of ED physician admission propensities across categories of patient sex, race and ethnicity, and Medicaid enrollment.

**Design, Setting, and Participants:**

This cross-sectional study analyzed Medicare fee-for-service claims for ED visits from January 1, 2016, to December 31, 2019, in a 10% random sample of hospitals. The allocation of patients to ED physicians in the acute care setting was used to isolate physician-level variation in admission rates that reflects variation in physician decision-making. Multi-level models with physician random effects and hospital fixed effects were used to estimate the within-hospital physician variation in admission propensity for different patient sociodemographic subgroups and the covariation in these propensities between subgroups (consistency), adjusting for primary diagnosis and comorbidities.

**Main Outcomes and Measures:**

Admission from the ED.

**Results:**

The analysis included 4 567 760 ED visits involving 2 334 361 beneficiaries and 15 767 physicians in 396 EDs. The mean (SD) age of the beneficiaries was 78 (8.2) years, 2 700 661 visits (59.1%) were by women, and most patients (3 839 055 [84.1%]) were not eligible for Medicaid. Of 4 473 978 race and ethnicity reports on enrollment, 103 699 patients (2.3%) were Asian/Pacific Islander, 421 588 (9.4%) were Black, 257 422 (5.8%) were Hispanic, and 3 691 269 (82.5%) were non-Hispanic White. Within hospitals, adjusted rates of admission were higher for men (36.8%; 95% CI, 36.8%-36.9%) than for women (33.7%; 95% CI, 33.7%-33.8%); higher for non-Hispanic White (36.0%; 95% CI, 35.9%-36.0%) than for Asian/Pacific Islander (33.6%; 95% CI, 33.3%-33.9%), Black (30.2%; 95% CI, 30.0%-30.3%), or Hispanic (31.1%; 95% CI, 30.9%-31.2%) beneficiaries; and higher for beneficiaries dually enrolled in Medicaid (36.3%; 95% CI, 36.2%-36.5%) than for those who were not (34.7%; 95% CI, 34.7%-34.8%). Within hospitals, physicians varied in the percentage of patients admitted, ranging from 22.4% for physicians at the 10th percentile to 47.6% for physicians at the 90th percentile of the estimated distribution. Physician admission propensities were correlated between men and women (*r* = 0.99), Black and non-Hispanic White patients (*r* = 0.98), and patients who were dually enrolled and not dually enrolled in Medicaid (*r* = 0.98).

**Conclusions and Relevance:**

This cross-sectional study indicated that, although overall rates of admission differ systematically by patient sociodemographic factors, an individual physician’s propensity to admit relative to other physicians appears to be applied consistently across sociodemographic groups of patients.

## Key Points

**Question:**

Are physician propensities to admit patients from the emergency department consistent across patient sociodemographic groups?

**Findings:**

In this cross-sectional analysis of Medicare claims data from 2016 to 2019, the mean adjusted rates of hospital admission from the emergency department differed by patient sex, race and ethnicity, and Medicaid status. Individual physicians varied substantially within hospitals in the percentage of patients they admitted, and these differences in admission propensity were consistent across patient sociodemographic groups.

**Meaning:**

The findings of this study suggest that physicians with higher or lower propensities to admit patients from the emergency department exhibit their relative propensity consistently across patient sociodemographic groups.

## Teaser

This cross-sectional study examines the consistency of variability in admission rates of patients presenting to the emergency department by sociodemographic factors.

## Introduction

Rates of inpatient hospital admission from the emergency department (ED) vary substantially across hospitals and regions even after controlling for patient comorbidities and hospital case mix.^1,2,3,4,5,6^ Variation in admission rates also exists for a wide spectrum of conditions across physicians within the same institution when variation in patient characteristics across physicians is minimal.^7,8,9,10^ It is not known, however, whether physician admission propensities are consistent for patients with different sociodemographic characteristics—that is, for example, whether physicians who tend to have high admission rates for patients who are White also have high admission rates for patients of other races and ethnicities.

Low consistency in physician admission propensities across sociodemographic groups would be suggestive, although not confirmatory, of individually mediated physician bias contributing to differential treatment by some physicians. However, high consistency would be less suggestive of individual biases because such individual biases are unlikely to be uniform in magnitude across all physicians.^11^

Disparities in health care access and outcomes based on race and ethnicity, sex, and socioeconomic status exist within the US health care system.^12,13^ In a nationally representative study, Zhang et al^14^ found that patients who were Black were 10% less likely to be admitted to the hospital from the ED compared with patients who were White. The extent to which differences in care reflect individually mediated clinician bias, whether conscious or not, is challenging to quantify and is often considered as a residual explanation after controlling for observable clinical factors and other patient differences attributable to structural factors, such as differences in health insurance. In this study, we sought to measure the consistency of variation in ED physicians’ admission propensities across Medicare patients who differ by race and ethnicity, sex, and Medicaid enrollment status.

## Methods

### Study Population and Data Source

We conducted a cross-sectional analysis of a sample taken from 100% of Medicare claims for ED visits from January 1, 2016, through December 31, 2019. We excluded ED visits made by Medicare Advantage enrollees as well as beneficiaries younger than 66 years and patients with end-stage kidney failure given the greater medical complexity and higher prevalence of disability resulting in distinctly higher ED use and admission rates.^15,16^ We restricted our analyses to nonsurgical *International Statistical Classification of Diseases, 10th Revision*, diagnoses over the study period. We further restricted the sample to a 10% random sample of hospitals with at least 5 physicians with at least 5 patient visits each. We excluded ED visits that occurred within 30 days of a previous visit (eMethods in the Supplement). This study was approved by the Harvard Medical School Committee on the Use of Human Subjects with a waiver of informed consent because of the use of a claims database. This study complied with the Strengthening the Reporting of Observational Studies in Epidemiology (STROBE) reporting guideline.

### Study Variables

Our outcome was hospital admission. For each ED visit, we determined whether the patient was discharged from the ED, admitted to the hospital, or admitted to observation status. Admission under observation status and transfers from the ED to another hospital were included in our sample and categorized as inpatient admissions (eMethods in the Supplement).

Independent variables of interest were patient race and ethnicity, recorded in the Medicare Beneficiary Summary File using the Research Triangle Institute (instead of the enrollment database) race variable as Asian/Pacific Islander, Black, Hispanic, non-Hispanic White, or other (including American Indian/Alaska Native), sex, and dual enrollment in Medicaid, which indicates low income among elderly beneficiaries.

As patient covariates, we also assessed patient age; primary diagnosis for the visit; visit day of week, month, and year of visit; and comorbidities, using both chronic disease indicators from the Chronic Conditions Data Warehouse and Hierarchical Condition Category scores.^17^

### Statistical Analysis

In previous work,^17^ we demonstrated that the distribution of patients to physicians within the same ED appears to emulate patient randomization to physicians with respect to observed patient characteristics. Thus, observed patient characteristics vary minimally across ED physicians within the same hospital on average, although this balance may not hold in all EDs or within an ED shift.^18^ In this study, we used this natural randomness to isolate physician-level variation in admission rates that was unrelated to variation in patient factors and thus mostly reflective of physician decision-making.^17^

We used a mixed-effects linear regression model to jointly estimate within-hospital physician-level variation and covariation in admission rates (unexplained by patient covariates) for each sociodemographic dimension along which patients were partitioned into subgroups (eg, male vs female; dual vs nondual Medicaid eligibility). For race and ethnicity, smaller sample sizes for the Hispanic and Asian/Pacific Islander subgroups, in part owing to underreporting of these subgroups in Medicare data,^19^ potentially impaired estimation of within-hospital physician-level covariation for those subgroups; thus, we focused on the more reliable estimates for patients who were Black or White and report estimates for other subgroups in the eMethods in the Supplement.

To facilitate interpretation, we report the physician-level covariation as a correlation coefficient for each pair of patient subgroups defined by the 3 sociodemographic characteristics of interest. The correlation coefficient is a derived parameter under the joint model, which estimates a variance-covariance matrix that captures the heterogeneity in physician admission decisions. We modeled hospital admission for a given ED visit as a function of hospital fixed effects, indicators of the sociodemographic subgroups, physician random effects for each subgroup (the term that yields the correlation coefficient of interest), patient covariates, and day of the week, month, and year of the visit. This modeling approach removes sampling errors from the estimation of physician-level variance and covariance parameters that would otherwise bias the correlation in physician effects between patient groups toward 0 (as reported in the eTable in the Supplement). We interacted all variables (including hospital fixed effects) with the subgroup identifiers (eg, male subgroup, female subgroup) and similarly allowed separate random effects for physician with each such subgroup; the latter also accomplishes the dual purpose of accounting for clustering within hospital and estimating a correlation between physician-level propensity for admission between the subgroups. We blocked rather than modeled hospital-level variation because our focus was on within-hospital physician-level variation. Statistical analysis was conducted using SAS version 9.4 (SAS Institute). The threshold for statistical significance was 2-sided *P* = .01.

## Results

Our analysis included 4 567 760 ED visits involving 2 334 361 beneficiaries and 15 767 physicians in 396 EDs. Mean (SD) age of the beneficiaries was 78 (8.2) years, and most were women (2 700 661 [59.1%]) vs men (1 867 099 [40.9%]). Of 4 473 978 reported indications on enrollment, race and ethnicity categories were Asian/Pacific Islander, 103 699 (2.3%); Black, 421 588 (9.4%); Hispanic, 257 422 (5.8%); and non-Hispanic White, 3 691 269 (82.5%). Most patients (3 839 055 [84.1%]) were not eligible for Medicaid. Patients in different sociodemographic groups differed in age, disability, and comorbidities but did not differ as much in the primary diagnosis for their ED visit (Table).

**Table.** Descriptive Summary of Overall Sample of Patient Characteristics

Variable	Race and ethnicity				Sex		Medicare/Medicaid eligibility	
	Asian/Pacific Islander (n = 103 699)	Black (n = 421 588)	Hispanic (n = 257 422)	Non-Hispanic White (n = 3 691 269)	Male (n = 1 867 099)	Female (n = 2 700 661)	Nondual (n = 3 839 055)	Dual (n = 728 705)
Age, mean (SD), y	79.2 (8.3)	76.6 (8.1)	77.5 (8.1)	78.7 (8.2)	77.5 (7.9)	78.9 (8.5)	78.0 (8.2)	77.9 (8.6)
Comorbidity indices, mean (SD)								
HCC score^a^	1.49 (1.37)	1.56 (1.56)	1.55 (1.48)	1.46 (1.36)	1.53 (1.47)	1.43 (1.32)	1.38 (1.30)	1.93 (1.70)
CCW score^b^	9.12 (4.07)	9.16 (4.09)	9.57 (4.41)	9.06 (3.98)	8.80 (4.11)	9.25 (3.97)	8.77 (3.95)	10.65 (4.11)
Most frequent diagnoses, No. (%)								
Nonspecific chest pain	6137 (5.9)	25 927 (6.2)	16 212 (6.3)	213 422 (5.8)	110 473 (5.9)	157 197 (5.8)	229 967 (6.0)	37 703 (5.2)
Abdominal pain	4674 (4.5)	17 837 (4.2)	13 272 (5.2)	145 990 (4.0)	67 845 (3.6)	118 145 (4.4)	156 894 (4.1)	29 096 (4.0)
Respiratory signs and symptoms	4306 (4.2)	17 603 (4.2)	9685 (3.8)	149 106 (4.0)	79 320 (4.3)	104 916 (3.9)	155 476 (4.1)	28 760 (3.9)
Superficial injury	3218 (3.1)	10 991 (2.6)	7703 (3.0)	142 568 (3.9)	59 675 (3.2)	108 013 (4.0)	142 561 (3.7)	25 127 (3.5)
Urinary tract infection	2991 (2.9)	13 600 (3.2)	9935 (3.9)	125 372 (3.4)	43 528 (2.3)	111 150 (4.1)	122 652 (3.2)	32 026 (4.4)
Musculoskeletal pain	2201 (2.1)	18 065 (4.3)	7924 (3.1)	105 800 (2.9)	45 422 (2.4)	91 314 (3.4)	115 257 (3.0)	21 479 (3.0)
Heart failure	2616 (2.5)	11 914 (3.1)	6476 (2.5)	96 398 (2.6)	55 932 (3.0)	64 457 (2.4)	99 298 (2.6)	21 091 (2.9)
Pneumonia	3301 (2.9)	8217 (1.9)	6198 (2.4)	95 648 (2.6)	53 802 (2.9)	61 561 (2.3)	92 180 (2.4)	23 183 (3.2)
Cardiac dysrhythmias	1820 (1.8)	5500 (1.3)	4027 (1.6)	101 053 (2.7)	48 940 (2.6)	65 632 (2.4)	103 011 (2.7)	11 561 (1.6)
Syncope	2669 (2.6)	10 862 (2.6)	4879 (1.9)	87 635 (2.4)	47 471 (2.5)	60 712 (2.3)	95 188 (2.5)	12 995 (1.8)
Medicaid status, No. (%)								
Nondual	47 401 (45.7)	296 148 (70.2)	136 368 (53.0)	3 285 423 (89.0)	1 626 324 (87.1)	2 212 731 (81.9)	NA	NA
Dual	56 298 (54.3)	125 440 (29.8)	121 054 (47.0)	405 846 (11.0)	240 775 (12.9)	487 930 (18.1)	NA	NA
Sex, No. (%)								
Male	47 401 (45.7)	157 902 (37.5)	99 834 (38.8)	1 520 656 (41.2)	NA	NA	NA	NA
Female	56 298 (54.3)	263 686 (62.5)	157 588 (61.2)	2 170 613 (58.8)	NA	NA	NA	NA

Abbreviations: CCW, Chronic Conditions Data Warehouse; HCC, Hierarchical Condition Category; NA, not applicable.

^a^ Higher HCC score signifies greater burden of chronic health conditions with no maximum achievable HCC score. In this sample the minimum was 0.29 and the maximum was 20.6.

^b^ Higher CCW score signifies greater burden of chronic health conditions with CCW score ranging from 0 to 27. The overall sample minimum was 0, and the maximum was 24.

Adjusted rates of admission were higher for men (36.8%; 95% CI, 36.8%-36.9%) than for women (33.7%; 95% CI, 33.7%-33.8%); higher for non-Hispanic White (36.0%; 95% CI, 35.9%-36.0%) than for Asian/Pacific Islander (33.6%; 95% CI, 33.3%-33.9%), Black (30.2%; 95% CI, 30.0%-30.3%), and Hispanic (31.1%; 95% CI, 30.9%-31.2%) (*P* < .001) beneficiaries; and higher for beneficiaries dually enrolled in Medicaid (36.3%; 95% CI,36.2%-36.5%) than for those who were not (34.7%; 95% CI, 34.7%-34.8%) (*P* < .001).

Within hospitals, physicians varied in the percentage of patients that they admitted from the ED, ranging from 22.4% of physicians at the 10th percentile to 47.6% of physicians at the 90th percentile of the estimated distribution. There was high correlation in physician admission propensities between men and women (*r* = 0.99), patients who were of Black and non-Hispanic White race (*r* = 0.98), patients who were of other racial and ethnic groups and those who were of non-Hispanic White race (eFigure in the Supplement), and those with and without Medicaid (*r* = 0.98), as estimated by our mixed-effect linear regression model (Figure).

**Figure.**
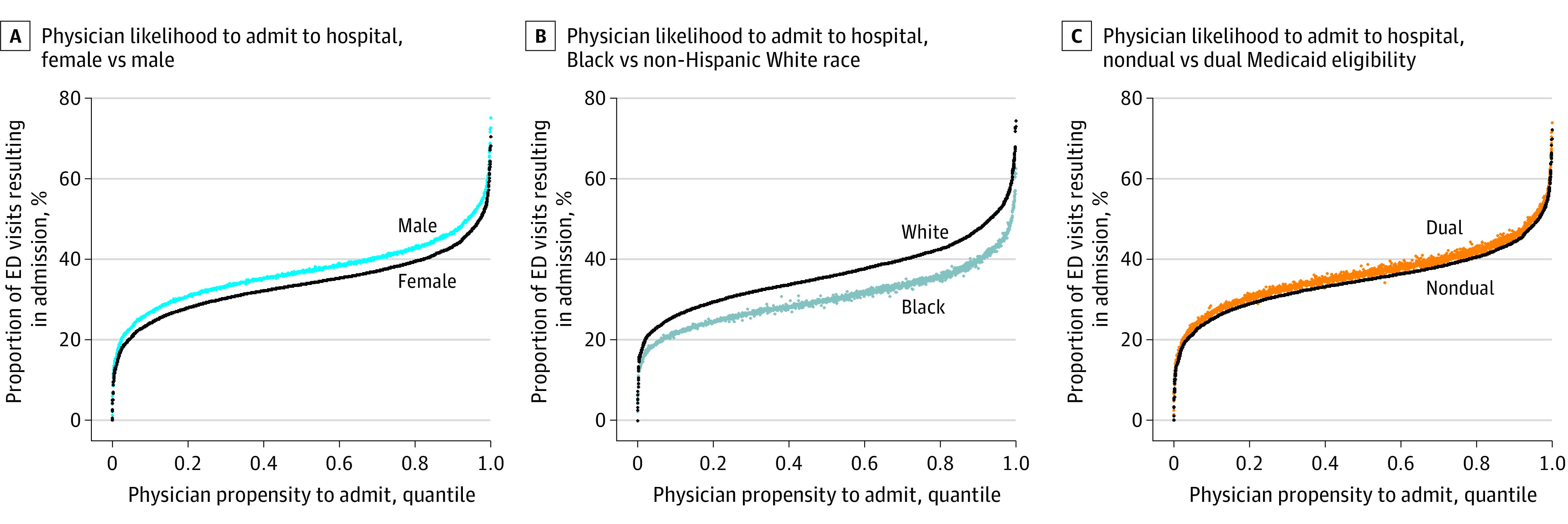
Distribution of Estimated Bivariate Physician Propensities to Admit Patients From the Emergency Department (ED) Admission from the ED shown by sex (A), race (B), and dual Medicaid eligibility (C). Adjustments were made for patient age; day of the week, month, and year of ED visit; visit diagnosis; and patient covariates. There is little noise in the admission rates for the populations other than for whom the data points are ordered (men, Black patients, and dually eligible patients), which signifies a significant correlation in physician admission propensities between subpopulations. The monotonic differences between the curves reflect the population-level differences in the subpopulation admission rates—not of differential heterogeneity between physicians.

## Discussion

We observed substantial overall differences in the rates of admission from the ED between Medicare patients of different sex, race and ethnicity, along with substantial variation across physicians in their overall propensities to admit. However, individual physician propensity to admit relative to other physicians appeared to be applied consistently across sociodemographic groups of patients. This finding suggests that physicians who are more or less likely to admit patients from the ED are more or less likely to do so regardless of the patient’s sex and race and ethnicity, or socioeconomic status (approximated by Medicaid enrollment status). Given that there was balance in observable patient characteristics across physicians within hospitals,^17^ we would not expect systematic sorting of patients at higher risk to some physicians as an explanation for the correlations we found.

Our study thus highlights an important distinction. Although there were differences in admission rates across patient sociodemographic groups, physicians’ relative propensities to admit patients from the ED were consistent across patient sociodemographic groups. Two explanations could plausibly reconcile the observed overall differences in admission rates between patient sociodemographic subgroups (eg, Black patients and women have the lowest admission rates) with the finding that physicians’ admission propensities were consistent across patient subgroups. One explanation is that all physicians share the same biases against admitting Black patients and women and exhibit this bias uniformly in magnitude. This explanation appears less likely because biases are likely to vary across physicians as documented in previous studies.^20^ An alternative explanation is that the overall within-hospital sociodemographic differences in admission rates that we observed are due to systemic or structural factors that affect patient sociodemographic subgroups differently.^21,22,23^ These systemic factors may include social variables impeding primary care access and resulting in higher ED use for less-severe presentations, insurance benefits or other income-related concerns about out-of-pocket costs, or mistrust of physician recommendations owing to a long history of racism and sexism in medicine and society.^24,25^

Although these factors are likely to vary across patients, our study design minimized patient differences among physicians by using the allocation of patients to ED physicians. Accordingly, patients’ exposures to outcomes associated with structural racism should theoretically distribute randomly across physicians within a hospital. In other words, within the limitations of our sampling and statistical methods, if differences in the outcomes of such systemic factors in different patient subgroups are held constant across physicians within EDs, as would be the case assuming random sorting, the consistency across ED physicians in the between-subgroup difference in admission rates may reflect the consistency of ED physician decision-making. The high level of consistency in our study suggests that differential treatment of subgroups by individual physicians does not sufficiently explain the sizeable overall sociodemographic differences in admission rates that we observed. Our findings may be important for policy and research because they suggest that efforts to address racial disparities should consider structural factors associated with health care disparities faced by historically marginalized patients.

### Limitations

This study has limitations. Owing to the limited granularity of information available in Medicare administrative claims data, we could not control for all patient clinical factors, such as vital signs in the ED or laboratory test results, that could be considered in the decision to admit patients. Similarly, we were limited in the ability to delineate patient socioeconomic status precisely. We did not control for physician characteristics, particularly demographic characteristics, owing to limitations in the availability of this information in administrative claims. Our findings may not be generalizable to other aspects of hospital care, given the degree of standardization of health care practices within emergency medicine (eg, chest pain pathways) that may diminish opportunities for individual clinician biases to affect decision-making with regard to admission. In addition, because our analysis focused on only 1 specific outcome—hospital admission—the findings cannot be generalized to other treatment decisions in which individually mediated physician biases may play more of a role.

## Conclusions

This cross-sectional study indicated that, although there are differences in the overall rates of admission by patient sociodemographic factors, an individual physician’s propensity to admit compared with other physicians appears to be applied consistently across sociodemographic groups of patients.

## Notes

Supplement. **eMethods**. Detailed Methods **eFigure.** Sensitivity Analysis of Joint Versus Stratified Mixed-Effect Regression Modeling of Hospital Admission **eTable.** Correlation in Physician Admission Tendency by Patient Sex Subgroup
