# Supplementary material for: Analysis of Consistency in Emergency Department Physician Variation in Propensity for Admission Across Patient Sociodemographic Groups
Source: JAMA Netw Open. 2021 Sep 21;4(9):e2125193. doi: 10.1001/jamanetworkopen.2021.25193 (PMC8456378; doi:10.1001/jamanetworkopen.2021.25193)
Supplement: Supplement. — eMethods. Detailed Methods eFigure. Sensitivity Analysis of Joint Versus Stratified Mixed-Effect Regression Modeling of Hospital Admission eTable. Correlation in Physician Admission Tendency by Patient Sex Subgroup [file jamanetwopen-e2125193-s001.pdf]

Supplementary Online Content

Khidir H, McWilliams JM, O'Malley AJ, et al. Analysis of consistency in emergency department physician variation in propensity for admission across patient sociodemographic groups. *JAMA Netw Open*. 2021;4(9):e2125193. doi:10.1001/jamanetworkopen.2021.25193

eMethods. Detailed Methods

eFigure. Sensitivity Analysis of Joint Versus Stratified Mixed-Effect Regression Modeling of Hospital Admission

eTable. Correlation in Physician Admission Tendency by Patient Sex Subgroup

This supplementary material has been provided by the authors to give readers additional information about their work.

© 2021 Khidir H et al. *JAMA Network Open.*

eMethods Detailed Methods

A. Sample selection

We started with 100% of Medicare claims for ED visits from January 1, 2016, through December 31, 2019. We identified ED visits and physicians caring for patients during these visits using Carrier claims (Healthcare Common Procedure Coding System (HCPCS) codes: "99281 – 99285, 99291, 99292, 99234-6, 99217-9, 99220, 99224-6" and Place of Service coded “23”) and the National Provider Identifier within carrier claims. We excluded ED visits made by beneficiaries who were under age 66 (to allow 1 full year of Medicare coverage), had Medicare Advantage, or had ESRD. We excluded ED visits that occurred in hospitals with less than 5 physicians who did not care for at least 5 patients. We excluded ED visits that resulted in a surgical ICD-10 diagnosis or ICD-10 diagnoses which had less than 50,000 visits over the study period. We also excluded revisits to the ED, defined as ED visits that occurred within 30 days of a prior ED visit, to avoid having duplicate disposition outcomes for the same clinical episode. After exclusion criteria were applied, we sampled 10% of hospitals represented and identified all non-surgical ED visits that occurred. For our statistical modeling, we restricted our sample to a random 30% sample of physicians.

B. ED visit disposition determination

For each ED visit, we determined whether the patient was discharged from the ED, admitted to the hospital, or admitted to “observation” status. Hospital transfers were included in our sample and categorized as inpatient admissions. ED visit disposition was determined by matching the identified visits from the Carrier file to inpatient and outpatient claims records. Observation visits were identified through billing codes for observation care in the Carrier file that were then

© 2021 Khidir H et al. *JAMA Network Open.*

matched to a specific ED visit using the date and the ED revenue center codes in the Outpatient file. Observation visits were considered as admissions for the purposes of our analyses.

C. Correlation in ED admission propensities for non-White Hispanic and Asian/Pacific Islander sample

Medicare administrative data undercount non-White Hispanic and Asian/Pacific Islander beneficiaries, limiting the completeness and accuracy of research studies that examine disparities in care related to these populations. Indeed, non-White Hispanic and Asian/Pacific Islander beneficiaries accounted for a very small percentage of ED visits within our sample period (5.75% and 2.32% respectively) compared to Black and White beneficiaries (9.42% and 82.51% respectively). Small sample sizes for the non-White Hispanic and Asian/Pacific Islander categories limit the accuracy our statistical model for those racial categories. Thus, given concerns regarding limitations in model accuracy, we have opted to report correlation coefficients for the two largest categories (Black and White) in the main text of our manuscript and provide the correlation coefficients for the two smallest categories (non-White Hispanic and Asian/Pacific Islander).

© 2021 Khidir H et al. *JAMA Network Open.*

eFigure. Sensitivity analysis of joint versus stratified mixed-effect regression modeling of hospital admission

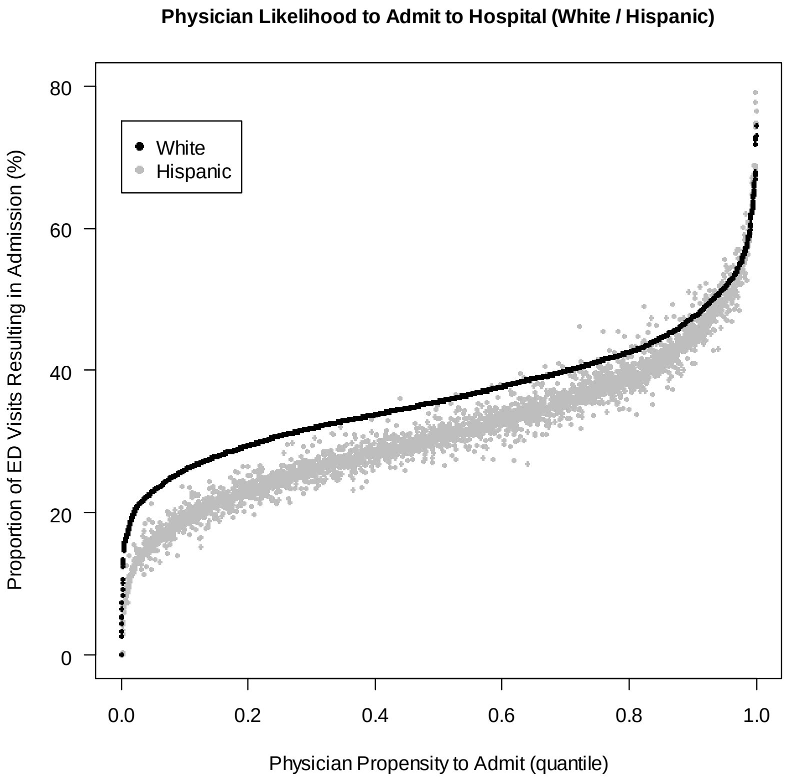

© 2021 Khidir H et al. *JAMA Network Open.*

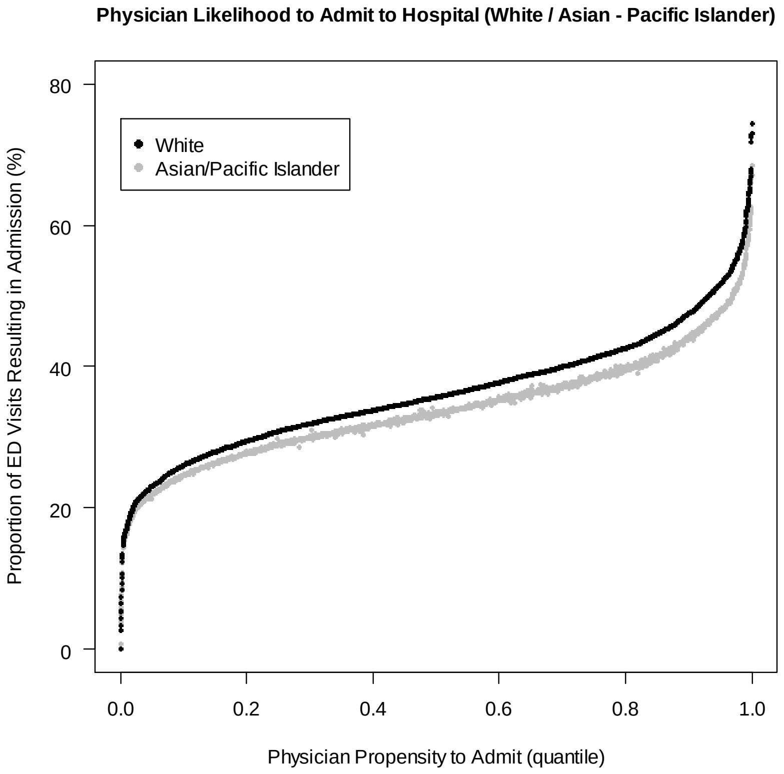

We estimated the correlation in physician admission tendencies between different sociodemographic groups of patients by fitting a multilevel model that jointly estimated the physician-level variance in each subgroup and the physician-level covariance. We implemented this joint modeling approach to estimate the physician-level correlation net of measurement error from physicians' having finite sample-sizes of ED visits. In contrast, if we first estimated separate models for each patient subgroup and then correlated the estimates of the physician effects, the resulting correlation would be biased towards zero ("attenuated"). (unpublished information)

Intuitively, attenuation bias is witnessed from the following thought experiment. Suppose that one subpopulation was huge (e.g., almost all of the ED visits) and the other was tiny (e.g., just 3 ED visits). The physician effects would be estimated with great precision for the large

© 2021 Khidir H et al. *JAMA Network Open.*

subpopulation and with minimal precision for the smaller subpopulation. Because the sample correlation would then be between a precisely measured variable and a quantity resembling random noise, the resulting correlation would be expected to be close to 0 even if the true correlation of the physician effects was close to 1. Because the joint model allows for uncertainty in the physician effects in accordance with the number of ED visits they saw, it implicitly accounts for the uncertainty in the physician effects when determining how correlated the true physician effects must be in order for the observed data to have resulted. Therefore, the estimates under the joint model are not compromised by attenuation bias.

Extending the above intuitive argument, as ED visit sample-sizes increase the physician estimates from the separate models will have smaller standard errors and the true physician effects will be better revealed allowing more of the true correlation to be revealed. However, only with infinite sample-sizes of ED visits or correlations that were exactly equal to 0 would the stratified approach yield estimates that correspond to those of joint model involving all physicians.

© 2021 Khidir H et al. *JAMA Network Open.*

eTable 1. Correlation in Physician Admission Tendency by Patient Sex Subgroup

	Correlation (male vs. female)
Stratified All Physicians	0.38
Stratified Top 50% of Physicians (N per subgroup= 90 ED visits per physician)*	0.42
Stratified Top 10% of Physicians (N per subgroup= 140 ED visits per physician)*	0.51
Stratified Top 5% of Physicians (N per subgroup= 164 ED visits per physician)*	0.54
Stratified Top 1% of Physicians (N per subgroup= 215 ED visits per physician)*	0.61
Joint Modeling All Physicians (correlation expected with infinite sample)	0.98

*N per subgroup assumes even distribution of male and female patient. ED visits to each physician.

In eTable 1, we demonstrate how our approach yields higher correlation estimates relative to naïve correlations produced by a stratified approach. We additionally demonstrate that the attenuation bias introduced by the stratified approach is partially ameliorated when restricting to physicians in top percentile of the sample (top 50%, top 10%, top 5%, and top 1%) in terms of the number of ED visits the physician bills in the sample. We expect the correlation obtained under the separate models to increase as the fraction of physicians retained decreases as the retained physicians all have more ED visits than the excluded physicians. We focus on patient sex as an illustrative case.

The stratified approach using the full sample of physicians produces correlations that are much lower than those we estimate with the joint modeling approach and report in the paper. As the standard errors of the estimated physician-effects fall in value from limiting the sample to physicians with successively larger samples of ED visits, the physician-level correlation estimates obtained under the stratification approach rise consistently. As predicted from the earlier intuitive argument, as the threshold for retaining physicians' becomes more stringent, the

© 2021 Khidir H et al. *JAMA Network Open.*

estimates of physician admission tendencies have smaller standard errors so the correlation evaluated on the estimates from the stratified models is closer to the correlation that is evaluated under the joint model (the correlation between the true values of the physician effects), which is what we ideally want to evaluate.

© 2021 Khidir H et al. *JAMA Network Open.*
